# Do Changes in Hemoglobin and C-Reactive Protein Levels in Patients With Hip Tuberculosis After Two Months of Treatment Predict Tubercle Bacilli Deletion?

**DOI:** 10.7759/cureus.32853

**Published:** 2022-12-23

**Authors:** Hoan Do Dang, Thanh Dao Xuan

**Affiliations:** 1 General Surgery, National Lung Hospital/Ha Noi Medical University, Ha Noi, VNM; 2 Orthopedics, Ha Noi Medial University Hospital/Ha Noi Medical University, Ha Noi, VNM

**Keywords:** anemia, tuberculosis prognosis, crp, hemoglobin, hip tuberculosis

## Abstract

Background and objective

Tuberculosis (TB) of the hip refers to the hip infection caused by tubercle bacilli. Treatment for hip TB includes anti-TB medications, surgery to remove joint abscesses, and orthopedic surgery. It is necessary to conduct tests to confirm that the tubercle bacilli have been eradicated following the treatments. In this study, we aimed to assess the change in hemoglobin and C-reactive protein (CRP) serum levels in patients with hip TB before and two months after receiving specific treatments. We sought to determine whether they are significant tests for the treatment prognosis of hip TB.

Methods

We employed a prospective cohort design for this study. It was conducted at National Lung Hospital, Hanoi, and involved 24 hip TB patients with intra-articular abscesses who were treated at the center during the period from October 2016 to October 2021. Blood hemoglobin, CRP serum level, and abscesses on hip MRI were assessed before and two months after treatments. Hemoglobin was examined by spectrophotometry, and CRP serum was measured using the immunoturbidimetric method.

Results

Before treatments, the average hemoglobin level in the patients was 11.48 ± 1.85 g/dl; the average CRP serum level was 63.53 ± 36.47 mg/l. After two months of treatments, the average hemoglobin level increased significantly to 13.22 ± 1.36 g/dl, while the average CRP level reduced significantly to 12.55 ± 11.34 mg/l. However, five cases displayed abnormal findings. These five individuals continued to have intra-articular abscesses.

Conclusion

In individuals who reacted well to the therapy, blood hemoglobin and CRP serum levels improved. Blood hemoglobin and serum CRP assays can be utilized to monitor outcomes in hip TB therapy.

## Introduction

Tuberculosis (TB) of the hip is a type of hip infection caused by tubercle bacilli, bacteria that reach the hip from the primary TB complex through blood, lymphatic, or adjacent routes [1]. Hip TB constitutes approximately 15-20% of all osteoarticular TB cases, and the hip is the second most common site of osteoarticular involvement after the spine [2].

Treatment for hip TB includes anti-TB medications, surgery to remove joint abscesses, and orthopedic surgery [1,2]. According to studies by Saraf and Tuli [1] and Babhukar and Pande [2], patients can choose one of the following three orthopedic surgical methods to treat TB of the hip: Girdlestone procedure, hip fusion, or total hip replacement. In recent years, total hip replacement has become the treatment of choice for restoring hip function [1]. Some researchers have suggested that orthopedic devices can be used in *Mycobacterium tuberculosis (M. tuberculosis)*-infected environments, but others have demonstrated that the bacteria can infect prosthetic joints [3-6]. In clinical practice, treating hip TB prior to hip replacement is often recommended to guarantee that tubercle bacilli are fully removed [7,8].

Typically, almost all tubercle bacilli are cleared in about 80% of the cases after the initial intensive phase of treatment (first two months). However, in some situations, such as cases in which patients cannot tolerate anti-TB medications, those that involve drug-resistant *M. tuberculosis*, and cases in which abscesses were not wholly removed through surgery, the tubercle bacilli can persist and continue to develop even after the intensive phase. Joint replacement would be hazardous in these circumstances. Therefore, adequate tests are necessary to confirm that the tubercle bacilli have been killed following the treatment. Monitoring the patient’s blood tests is one of the prognostic methods for lung TB [9-12]. If the patient has recovered from anemia and the serum C-reactive protein (CRP) level is significantly reduced, then the results indicate that the tubercle bacilli have been eradicated.

The aim of the present study was to evaluate the changes in hemoglobin and CRP levels in patients with hip TB from the time of diagnosis to two months after receiving specific treatments and to compare the results with MRI scans of the hip. We tried to see if our findings supported the use of blood tests to monitor treatment and make a prognosis for hip TB outcomes.

## Materials and methods

For this prospective cohort study, we enrolled 24 patients with hip TB who received treatment at National Lung Hospital, Hanoi, between October 2016 and October 2021. The inclusion criteria were as follows: patients aged 20-80 years, with a diagnosis of hip TB, confirmed hip abscesses on MRI, and who gave consent to participate in the study. Exclusion criteria included patients with coinfection with other bacteria and those with an acute illness.

All patients with microbiological evidence of TB were treated with anti-TB drugs according to World Health Organization (WHO) guidelines. Surgical removal of joint abscesses was done following anti-TB drug treatment. We collected data on blood hemoglobin levels, serum CRP levels, and MRI of the pathologic hip prior to and two months after treatments to determine whether hemoglobin and CRP tests might predict the resolution of a hip abscess. Demographic characteristics, including age and sex, were also recorded.

The spectrophotometric method was used to test hemoglobin levels in the blood. Hemoglobin levels below 13 g/dL for men and 12 g/dL for women indicated anemia, which was classified into mild, moderate, and severe categories. Mild grade corresponded to a hemoglobin level of 11.0 g/dL to a lower limit of normal, moderate grade corresponded to a hemoglobin level of 8.0-11.0 g/dL, and severe grade corresponded to a hemoglobin level lower than 8.0 g/dL.

The immunoturbidimetric method was used to measure CRP. CRP levels greater than 10 mg/L were considered abnormal. Elevated CRP levels can indicate an infection, autoimmune diseases, inflammatory reactions, or tumors, among other conditions.

We performed a hip MRI to evaluate the hip abscess. On an MRI, an abscess appears as a distinct cavity filled with pus; it may also form a fistula into the femoral triangle or the rectal fossa or leak out into the skin. It needs to be distinguished from joint effusion on T1-weighted, T2-weighted, and diffusion-weighted imaging.

Statistical analysis

Data were managed and analyzed using IBM SPSS Statistics version 22.0 (IBM Corp., Armonk, NY). We used sample t-tests (two-tailed) and ANOVA to compare the means. A p-value <0.05 was considered statistically significant.

Ethical consideration

The authors are accountable for all aspects of the work by ensuring that questions related to the accuracy or integrity of any part of the work are appropriately investigated and resolved. The patients were informed in detail about the study and written informed consent was obtained. The study proposal was submitted to and approved by the Institutional Review Board of Hanoi Medical University (No. 69/GCN/HĐĐĐNCYSH-ĐHYHN, dated: March 17, 2020).

## Results

Demographic characteristics of participants

The study included 24 patients with hip abscesses due to TB. Most of them were men (19, 79.2%), and the most common age range was 41-60 years (n=11, 45.8%) (Table 1).

**Table 1:** Demographic characteristics

Characteristics	Frequency	Percentage
Sex		
Male	19	79.2
Female	5	20.8
Age, years		
20-40	7	29.2
41-60	11	45.8
61-80	6	25

Blood hemoglobin and serum CRP levels before treatment

Most patients had mild to moderate anemia (n=19, 79.2%), with an average hemoglobin level of 11.48 ± 1.85 g/dL for all patients. There was no statistically significant difference in mean hemoglobin levels by sex or age group before treatment (p>0.05). Similarly, most patients had an abnormally high CRP level prior to treatment (n=23, 95.8%), with a mean CRP level of 63.53 ± 36.47 mg/L for all patients. There was no statistically significant difference in mean CRP before treatment by sex or age group (p>0.05) (Table 2).

**Table 2:** Blood hemoglobin and serum CRP levels before TB treatment based on sex and age SD: standard deviation; CRP: C-reactive protein; TB: tuberculosis

Characteristics	Hemoglobin, g/dL, mean ± SD	P-value	CRP, mg/L, mean ± SD	P-value
Sex				
Male	11.12 ± 1.58	0.603	66.79 ± 40.14	0.160
Female	11.57 ± 1.94		51.14 ± 12.43	
Age, years				
20-40	10.52 ± 1.53	0.445	70.20 ± 43.90	0.438
41-60	11.74 ± 1.71		69.67 ± 33.87	
61-80	11.71 ± 2.28		63.53 ± 36.47	

Changes in hemoglobin and CRP levels after two months of treatment and their correlation with hip abscess resolution

The blood hemoglobin level increased significantly after two months of treatment (p<0.05), averaging 13.22 ± 1.36 g/dL compared with 11.48 ± 1.85 g/dL prior to treatment. However, five patients (20.83%) remained anemic. An MRI of the hip revealed that these five patients continued to have intra-articular abscesses. Similarly, after two months of treatments, serum CRP levels decreased significantly (p<0.05), with an average of 12.55 ± 11.34 mg/L compared with the pretreatment mean of 63.53 ± 36.47 mg/L. CRP levels in the majority of patients were less than 10 mg/L. However, five patients had CRP levels above 10 mg/L. These were the same ones who still had anemia and intra-articular abscesses (Table 3).

**Table 3:** Change of blood hemoglobin and serum CRP levels from before to two months after treatment CRP: C-reactive protein

Patients	Hemoglobin, g/dL		CRP, mg/L		Abscess
	Before treatment	Two months after treatment	Before treatment	Two months after treatment	Two months after treatment
1	11.20	11.40	52.00	18.20	+
2	10.60	11.00	66.20	22.10	+
3	12.80	13.80	42.20	8.00	−
4	9.900	10.40	39.20	25.20	+
5	14.00	14.00	59.20	6.00	−
6	11.40	13.20	65.20	7.20	−
7	8.50	13.40	75.10	9.10	−
8	15.00	15.00	122.10	8.20	−
9	9.20	13.20	135.20	3.20	−
10	9.60	14.20	65.20	7.20	−
11	10.10	13.20	38.20	5.40	−
12	13.10	13.00	87.50	8.20	−
13	12.40	13.80	69.80	6.30	−
14	9.70	10.20	38.20	25.00	+
15	11.30	13.60	150.00	7.20	−
16	11.60	14.30	52.80	5.90	−
17	13.80	14.20	60.10	5.40	−
18	13.10	14.20	36.80	22.10	−
19	12.70	15.10	35.00	51.40	−
20	12.70	14.10	122.20	4.30	−
21	13.40	13.20	35.50	8.40	−
22	8.100	13.20	3.00	3.20	−
23	11.30	11.40	50.10	27.80	+
24	10.00	14.10	23.90	6.10	−
Average	11.48 ± 1.85	13.22 ± 1.36	63.53 ± 36.47	12.55 ± 11.34	N/A
P-value	0.001		<0.001		N/A

## Discussion

Before treatment, the majority of patients with a hip TB abscess had mild to moderate anemia, with an average hemoglobin of 11.48 ± 1.85 g/dL. The severity of anemia was unrelated to age or sex. These findings are consistent with those of Dasaradhan et al. [13], Chhabra et al. [14], and de Mendonça et al. [15], who reported that anemia occurs in 61.53-85.7% of TB patients. Patients are anemic because they are malnourished due to the energy expended on combating TB. In addition, TB causes loss of appetite, thereby reducing the energy income in the patient’s body. Furthermore, TB reduces ferritin absorption, resulting in microcytic anemia [9]. Hence, although a patient may have an appropriate number of red blood cells, their hemoglobin level is still low; thus, we used a hemoglobin test rather than a red blood cell count to assess TB anemia more reliably.

After two months of treatment, the tubercle bacilli causing the hip abscess disappeared and did not reappear in the follow-up. We used the BACTEC method to detect *M. tuberculosis *before orthopedics surgery and got a negative result in cases without abscesses. This disappearance was also reflected in the patient’s recovery from anemia, with hemoglobin levels returning to normal.

In addition to the hemoglobin test, CRP testing is crucial for monitoring hip TB. Some studies, such as those by Soedarsono and Subiantoro [16] and Wilson et al. [12], showed that CRP levels in TB patients were substantially higher (average levels of 64.8 mg/L and 125 mg/L, respectively). In our study, CRP levels were increased in patients with hip TB, with an average CRP of 63.53 ± 36.47 mg/L, but they declined considerably after two months of treatment, reaching 12.55 ± 11.34 mg/L on average. Following the response to treatment, the CRP level fell significantly.

Analyzing each patient in our research group, we discovered that even after two months of treatment including TB medicines and surgical elimination of abscesses, five individuals still had joint abscesses on MRI. All five of these individuals remained somewhat anemic, with hemoglobin levels averaging 10.88 g/dL and CRP levels averaging 23.66 mg/L. As a result, we conclude that blood hemoglobin and serum CRP are functional diagnostics for monitoring hip TB therapy. Because some patients with hip TB may be anemic or have raised CRP levels, hemoglobin and CRP tests should be integrated into clinical practice to monitor TB treatment.

Limitations

Because the study only included 24 patients with hip TB, it could not investigate other comorbidities associated with anemia and CRP elevation, such as pulmonary TB, systemic disease, gout, and rheumatoid arthritis.

## Conclusions

Patients with TB of the hip joint had anemia and increased serum CRP levels prior to treatment. After two months of treatment, blood hemoglobin and serum CRP levels returned to normal, and the intra-articular abscess disappeared. Patients who did not respond favorably to therapy remained anemic and continued to have elevated CRP levels. These results suggest that blood hemoglobin and serum CRP testing can be used to monitor and evaluate hip TB treatment.
